# Association between moderate-to-vigorous physical activity and attention among children aged 6–12 years: chain mediating effects of fundamental movement skills and aerobic fitness

**DOI:** 10.3389/fped.2024.1451662

**Published:** 2024-10-31

**Authors:** Haitan Wu, Xidong Wang, Zhangyi Jin

**Affiliations:** School of Physical Education, Shanghai Normal University, Shanghai, China

**Keywords:** moderate-to-vigorous physical activity, fundamental movement skills, aerobic fitness, attention, children

## Abstract

**Purpose:**

To explore the association between physical activity and attention among children aged 6–12 years and to determine whether fundamental movement skills and aerobic fitness mediate the association between physical activity and attention.

**Methods:**

A total of 1,086 school-aged children (mean age: 9.40 ± 2.03 years) were included in the present study. Physical activity was assessed using the International Physical Activity Scale-Short Form. Fundamental movement skills were assessed using the Test of Gross Motor Development, third edition, and aerobic fitness was evaluated using the 20-metre shuttle run. Attention was assessed by the d2 Attention Test.

**Results:**

There were significant positive correlations between physical activity and fundamental movement skills, physical activity and aerobic fitness, and physical activity and attention (all *p* < 0.05). Both fundamental movement skills and aerobic fitness fully mediated the relationship between physical activity and attention. In addition, fundamental movement skills and aerobic fitness had chain-mediating effects on the relationship between physical activity and attention.

**Conclusion:**

High levels of physical activity were positively associated with attention among school-aged children. Fundamental movement skills and aerobic fitness played a chain-mediating role in the relationship between physical activity and attention.

## 1 Introduction

Attention is crucial in the development of adolescents and children and is generally considered an important prerequisite for successful adolescent learning (1). Attention is a cognitive process that is essential for learning and movement (2, 3). Moreover, it is the primary task of children's growth and helps improve cognitive function (4). Attention is directly related to mechanisms such as perception, memory, and executive function. In the learning process and in social psychological adjustment, attentional control reflects the higher cognitive functions of the brain (5). It acts as a “gateway” to working memory, mediating the flow of sensory information into consciousness (6), as measured by reaction time or performance on other simple decision-making tasks (7). For juveniles, attention plays an important role in the selection of some available perceptual information (8). Insufficient attention is associated with poorer academic performance (9). In addition, some studies have shown that physical activity has relatively little impact on information processing, reaction time, and memory but has a greater impact on attention (10). Existing evidence shows that physical activity not only affects the cognitive function of adults but also affects children and adolescents (11, 12). However, most related studies have focused on overall cognitive function, and few studies have reported the specific association between cognitive function and attention. In addition, few studies have focused on the mediating mechanisms behind physical activity and attention among school-aged children.

### 1.1 Aerobic fitness and attention

According to Tomporowski (13), Best (14) and Pesce (15), physical activity research has focused on the “cardiovascular health hypothesis” of the potential mechanisms of cognitive function that have positive effects. Although, the “cardiovascular fitness hypothesis” has not been supported by a systematic examination of the relationship between aerobic fitness and cognitive function, cross-sectional studies have consistently shown that healthy adolescents exhibit better cognitive function than unhealthy adolescents aged (16–18).

Recent studies regarding the association between aerobic fitness and attention have focused particularly on children because childhood is a critical period for brain development. Research has shown that higher aerobic fitness levels are associated with an improved ability to perform cognitive tasks requiring variable cognitive demands (19, 20). Relevant experimental research has shown that high aerobic fitness promotes better performance on cognitive control tasks (21). Aerobic fitness also influences development of new blood vessels (angiogenesis) and neurons (neurogenesis) and promotes increased synaptic plasticity in brain areas that support various cognitive functions (14). Furthermore, performing regular exercise at aerobic intensities is positively associated with cognitive tasks (22). Increased levels of aerobic fitness lead to increased cognitive function and improved brain health (23, 24).

Most studies on the relationships between physical and cognitive factors have focused on the associations between cardiovascular aspects of physical fitness (also termed “aerobic fitness”) and academic performance (25, 26), executive function (17, 21, 27), or working memory (28). However, few studies have examined the association between aerobic fitness and attention among school-aged children.

### 1.2 Fundamental movement skills and attention

The interconnectedness of motor development and cognitive development has underpinned fundamental theories in child development. Related research has shown a significant positive relationship between motor skills and executive function at a global level as well as at a specific level of analysis. According to “dynamic systems theory”, movement is produced as a result of interactions between subsystems, such as the cognitive, neurological, muscular and skeletal systems (29), and relevant reports have indicated that motor skills influence cognitive development (30). Reciprocity occurs when fundamental movement skills and executive functions develop and improve alongside each other (31).

Both theoretically and empirically, there is continuous interest in understanding the specific relation between fundamental movement skills and cognition in childhood. Research has shown that a person should acquire and develop the ability to regulate attention, working memory, flexibility, and executive functions starting in childhood (32). Children's movement skills improve with extended physical activity and motor training (33), and those with better fundamental movement skills are predicted to have better attention. Studies of children with the inattentive subtype of attention deficit and hyperactivity disorder (ADHD) seem to present more impairment of fine motor skills, slow reaction time, and online motor control during complex tasks (34). Explaining the correlation between fundamental movement skill development and attention. Previous studies have supported this interrelation, focusing primarily on fundamental movement skills, cognition (35) and overall executive function performance (36, 37, 38). However, there are currently few reports on the relationship between fundamental movement skills and attention in children.

### 1.3 Physical activity and attention

Attention in young children benefits most from physical activity targeting fundamental movement skills and aerobic fitness (39), which researchers have given particular attention to it (10). To some extent, learning motor skills can enhance social interactions or increase cognitive participation, which may affect the cognitive function of children. Studies have shown that physical activity-induced cardiovascular health can cause changes in the relationship between physical activity and cognitive function through increased mediation (40). Although the findings appear to be conflicting, and while the discrepant results found in cross-sectional studies could be explained by potentially confounding variables, such as differences in education or nutritional habits, the effects demonstrated by the above intervention studies do not necessarily account for the causal effect of enhanced aerobic fitness; in some cases, the subjects may also be affected by other relevant factors in the physical activity process. These interventions include not only aerobic fitness training but also a variety of activities, such as games, ball exercises, or other programs.

The ability of physical activity to improve the cognitive function of children is affected by many factors. A physical activity that requires various motor skills and is suitable for children is more in line with the requirements of physical education courses than is pure aerobic physical training. Recent research has shown that targeted physical activity interventions have positive effects on children's cognition (41, 42), while attempts to systematically use challenging cognitive physical activity (learning content that incorporates motor skills) to boost cognitive function have rarely been detected (43, 44); moreover, there are few reports on related research.

“Variables other than aerobic fitness” deserve attention when studying the relationship between physical activity and cognitive function. Furthermore, a sufficient level of moderate-vigorous physical activity can increase aerobic fitness (45), which might be a prerequisite for improving executive function (the cardiovascular fitness hypothesis) (46). Research has shown that children with ADHD can seemingly demonstrate a high level of physical activity, and a frequent prevalence of motor deficits as well as an overall low level of motor competence are confirmed (47).

To date, no study has focused on the association between physical activity and attention in children. Exploration of the relationship between physical activity and attention in children is desirable and necessary due to the lack of consistent and convincing evidence.

### 1.4 Research aims

**Figure 1:**
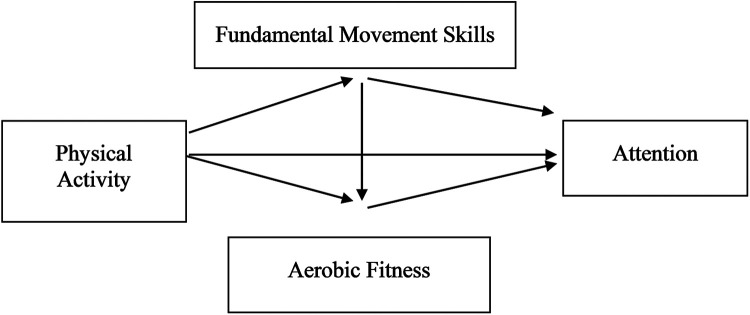
Hypothetical model diagram.

In summary, the effects of physical activity on attention may be positively influenced by aerobic fitness and fundamental movement skills. This study explored the relationship between physical activity and attention from two aspects—fundamental movement skills and aerobic fitness—and investigated the role of fundamental movement skills and aerobic fitness in physical activity and attention. The research adopted questionnaires and test methods, considered physical activity and fundamental movement skills as independent variables and attention as the dependent variable, and investigated the mediating effect of fundamental movement skills between physical activity and attention. Moreover, we further explored the chain mediating effect of fundamental movement skills → aerobic fitness. The research hypotheses were as follows: (1) physical activity is significantly positively correlated with fundamental movement skills, aerobic fitness, and attention; (2) fundamental movement skills play a mediating role between physical activity and attention; (3) aerobic fitness plays a mediating role in the relationship between physical activity and attention; and (4) fundamental movement skills and aerobic fitness play a chain mediating role in the relationship between physical activity and attention. A diagram of these hypotheses is shown in Figure 1.

## 2 Methods

### 2.1 Participants

In this study, a cluster sampling method was used to select students from Grade 1 to Grade 5 from 6 schools for inclusion in the study. The average age of the 531 boys and 555 girls included was 9.40 ± 2.03 years. A total of 1,162 questionnaires were distributed, 1,160 were recovered, and 1,086 were valid, resulting in an effective response rate of 93.5%. Before the questionnaires were issued, the rules for completing the questionnaires were be explained to all participants in the same manner, and the distribution and recycling were carried out consistently.

### 2.2 Physical activity

The International Physical Activity Questionnaire (IPAQ) was developed by the World Health Organization in 1998 and is recommended as a cost-effective and widely used scale for evaluating moderate-to-vigorous physical activity. This study used the Chinese version of the short questionnaire, which comprises a total of 7 questions, and it took approximately 5 min for individuals to complete the questionnaire (48), with an overall moderate risk of bias and a total score of 0.43 (49).

### 2.3 Attention

The d2 Attention Test is an international authoritative test (50), and the d2 Attention Test compiled by Brickenkam et al. (1998) was used mainly in this study to measure the selective attention ability of individuals. The test items consisted of “d”, “p” and 1–4 short lines, which appeared on the upper or lower part of “d” or “p” alone or in pairs. In the test, participants were asked to look carefully at each line of characters, identifying and crossing out all “d”s with two dashes. The participants were required to complete a line within 20 s, and the test results were scored using a manual scoring system. The index of the test used in this study was the total score (TN-E), which is calculated as the total number of processed items divided by the total number of processed items (TN) minus the number of errors (E1 + E2) (E1 represents missing responses and E2 represents wrong choices). This metric can be used to measure the relationship between attentional control and inhibitory control, as well as the speed and accuracy of the operation process. The scale has good theoretical validity, construct validity, internal consistency reliability and test-retest reliability and can be used as a reliable and effective research tool for selective attention.

### 2.4 Fundamental movement skills

The fundamental movement skills included in the Test of Gross Motor Development (TGMD-3) compiled by Dale A. Ulrich of the University of Michigan were run, gallop, hop, skip, horizontal jump, and slide. The holding skills included two-hand strike, one-hand strike, dribble, overhand throw, underhand throw, catch, and kick. There were 13 items in total (51).

TGMD-3 test indicators, according to the test requirements, were video shooting and the admission materials, and the final video involved 9 personnel, including subject experts, physical education experts, and postgraduate members. These participants were scored according to the TGMD-3 test scale and scores were tallied over 3 rounds. After 12 h of training, the raters’ back-to-back scoring consistency reached more than 90% and scores were consistent among scored groups. Additionally, standardized data processing was performed on the motor skills videos of each child.

### 2.5 Aerobic fitness

The FITNESSGRAM test was used, and a 20-metre shuttle run test (20 m SRT) was selected for the test (52). All the testers were provided with the same training according to the test requirements. The number of completed tracks (i.e., the number of times a child ran back and forth) was used as a score of aerobic endurance.

### 2.6 Investigation procedure

After school principals provided consent, the parents and students signed informed consent forms, and the test was administered by uniformly trained test personnel. The principals of all the schools granted permission for students in their schools to participate in this study. The university's Institutional Review Board approved the study protocols (SHNU2022070). The study requirements were explained and questions were answered on the spot. When the students fully understood the feedback, and after they completed the questionnaire.

### 2.7 Data collection

Members of the research team explained and demonstrated how to complete the International Physical Activity Questionnaire (IPAQ), d2 test, TGMD-3 and 20 m SRT based on the standardized testing directions. Then, the students were asked to practice the test provided with the standardized test directions to ensure that all the participants understood the testing procedures. The research period started in September 2020 and ended in June 2023.

### 2.8 Statistical analysis

Data processing was carried out using SPSS 24.0 and AMOS 24.0. First, the data were checked and cleaned, the missing data were checked for randomness, and the missing data were found to be completely missing at random. When there was less than 2% of the data missing for the main variable, the missing data were replaced by the mean value. According to Harman's suggestion, the single-factor test method was used to analyze common method bias, and exploratory factor analysis revealed that the variance explained by the first unrotated factor was 31.28%, which was less than the critical value of 40%, indicating that there was no obvious difference in this study. Finally, correlation analysis was performed, and based on the hypothetical model, physical activity was used as the predictor variable, attention was used as the outcome variable, and fundamental movement skills were used as the mediator variable for path analysis. A latent variable structural equation model was used to test the hypothesized direct effect model and mediation effect model.

## 3 Results

### 3.1 Correlations among physical activity, fundamental movement skills, aerobic fitness and attention

The average time the children and adolescents spent engaged in physical activity was 3,287.48 ± 2,395.33 min/week. The scores for locomotor skills, object control skills, and aerobic fitness were 30.85 ± 5.99, 34.39 ± 7.37, and 19.92 ± 11.69 points, respectively. The average attention score was 301.49 ± 110.24 points. Correlation analysis revealed significant positive correlations between all variables. For example, physical activity level was positively correlated with attention (*r* = 0.097, *p* < 0.05), aerobic fitness (*r* = 0.204, *p* < 0.01), locomotor skills (*r* = 0.082, *p* < 0.01), and object control skills (*r* = 0.202, *p* < 0.01) (Table 1).

**Table 1:** Correlation analysis of physical activity, fundamental movement skills, aerobic fitness and attention.

	1	2	3	4	5	6
1 PA level	1					
2 PA time	.637^a^	1				
3 Locomotor skills	.082^b^	.090^a^	1			
4 Object control skills	.202^a^	.163^a^	.499^a^	1		
5 Aerobic fitness	.202^a^	.160^a^	.392^a^	.474^a^	1	
6 TN-E	.097^b^	.018	.206^a^	.214^a^	.317^a^	1

PA, physical activity.

^a^ *p* < 0.01.

^b^ *p* < 0.05.

### 3.2 The mediating role of fundamental movement skills between physical activity and attention

**Figure 2:**
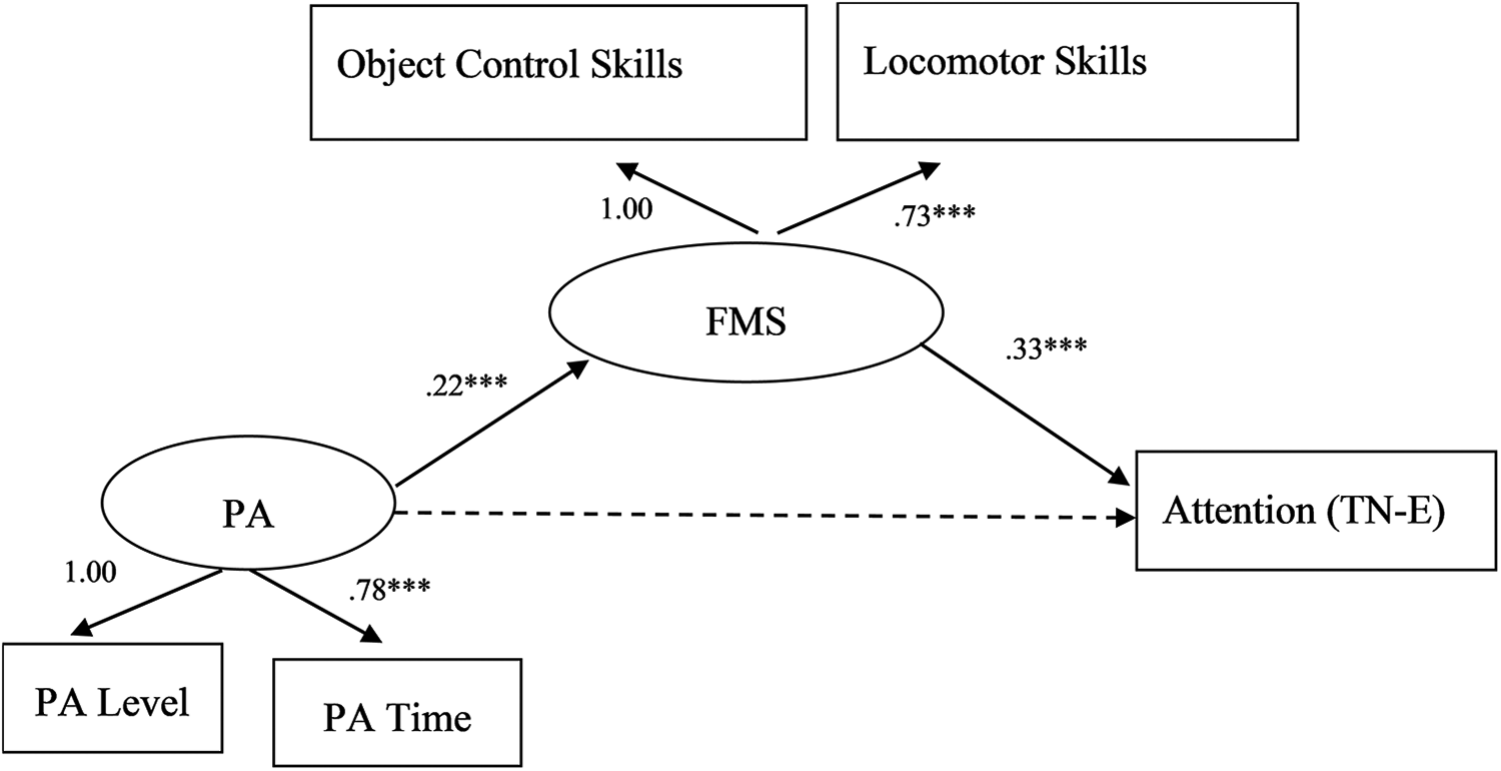
The mediating role of fundamental movement skills in physical activity and attention. **p < 0.01, ***p < 0.001. The path coefficients listed in the figure are standardized coefficients.

Physical activity had a positive predictive effect on attention (*b* = 0.03, SE = 0.04, *p* = 0.45); the data fitting results are shown in Figure 2. For the data shown in Figure 2, χ^2^/df = 5.992, CFI = 0.985, TLI = 0.950, NFI = 0.982, GFI = 0.994, RMSEA = 0.068, and RMR = 0.023. All the fitness statistics were within a reasonable range, and the results of the analysis supported the rationality of the preliminary model constructed in this study.

From the perspective of the model path, children's physical activity cannot directly and positively predict attention but indirectly predicts attention through fundamental movement skills (standardized regression coefficient = 0.22 × 0.33 = 0.0726). Fundamental movement skills fully mediated the relationship between physical activity and attention. The 95% confidence interval (CI) of the indirect effect of “physical activity → fundamental movement skills → attention” was [0.035, 0.129], and the mediation effect size was 71.84%. The CI does not include 0, indicating that the mediating effect of fundamental movement skills is statistically significant. In addition, these skills play a complete intermediary role.

This study's findings are similar to those of previous studies. Fundamental movement skills mediate the relationship between physical activity and attention, which supports the effects of physical activity on attention. Physical activity affects the development of students’ attention through the mediating effect of fundamental movement skills. These skills have a facilitative effect, indicating that fundamental movement skills are an important mediator of physical activity-promoted attention improvement.

### 3.3 Chain mediation of fundamental movement skills and aerobic fitness between physical activity and attention

**Figure 3:**
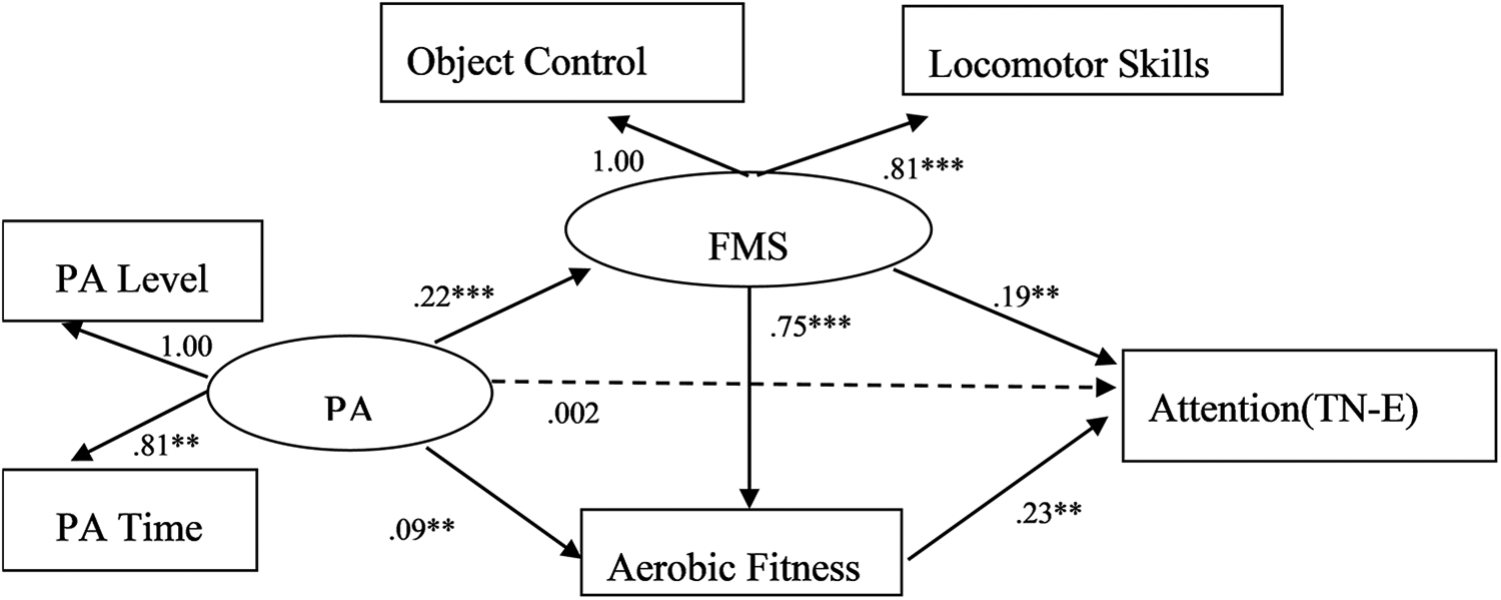
A multiple mediating model of physical activity predicting attention. **p < 0.01, ***p < 0.001. The path coefficients listed in the figure are standardized coefficients.

Structural equation modelling was further used to examine the chain mediating effect of fundamental movement skills and aerobic fitness on the relationship between physical activity and attention (Figure 3). The observed data fit the hypothetical model well (χ^2^/df = 3.772, CFI = 0.990, TLI = 0.970, NFI = 0.987, GFI = 0.994, RMSEA = 0.051, RMR = 0.020). The chain mediation test showed that physical activity can positively predict fundamental movement skills (*r* = 0.220, *p* < 0.001), fundamental movement skills can positively predict aerobic fitness (*r* = 0.755, *p* < 0.001), and aerobic fitness can positively predict attention (*r* = 0.226, *p* < 0.001); that is, the more physical activity a person engages in, the greater the level of fundamental movement skills, which in turn promotes greater improvement in aerobic fitness and ultimately improves attention.

Using bias-corrected percentile bootstrapping to test the mediating effect, the 95% range of the mediating effect of fundamental movement skills on physical activity and attention was [0.016, 0.086], the mediating effect size was 41.18%, and the mediating effect was significant. Therefore, fundamental movement skills are the intermediate variable between physical activity and attention, again verifying hypothesis 2. The 95% CI of the mediating effect of aerobic fitness on physical activity and attention was [0.007, 0.042], the mediating effect size was 19.61%, the complete mediating effect was significant, and hypothesis 3 was verified. The 95% CI of the chain mediating effect of fundamental movement skills and aerobic fitness between physical activity and attention was 0.020 (0.064), and the mediating effect size was 37.25%. The chain mediating effect was significant, and hypothesis 4 was verified. Thus, fundamental movement skills and aerobic fitness act as chain mediators between physical activity and attention.

## 4 Discussion

Relevant studies have shown that the relationship between physical skills, such as throwing and kicking speed, and health-related physical fitness gradually increases as children age, and children should focus on developing motor skills and enhancing health-related physical fitness (53). Concentration is an important prerequisite for learning (54) and has high long-term predictive validity for children's academic performance (55). Because of its relevance to the entire learning process in school, ecologically feasible interventions are often required to promote attention in children (56).

Related studies have shown that physical activity has a positive effect on children's attention (32, 35, 50). A recent meta-analysis by Fedewa and Ahn (57) showed that physical activity has a positive effect on children's cognitive ability or academic achievement (the effect size was 0.28), and regular exercise had the greatest improvement effect (58). Another study showed that the enhancement of cognitive function brought about by physical activity is most obvious in attention (3). According to related studies, the link between physical activity and attention can be hypothesized by different underlying neuro-physiological and psychological mechanisms. These short-term effects can be explained by increased cerebral blood flow (59); increased release of various neurotrophic factors, such as brain-derived neurotrophic factor (BDNF) or nerve growth factor (60, 61, 62); elevated levels of glucocorticoids, such as cortisol (63); and catecholamine release of epinephrine, epinephrine or dopamine (62). These neurophysiological changes, particularly elevated catecholamines, are thought to in turn lead to altered mental states, such as increased arousal, increasing the availability of attentional resources and thus promoting performance on cognitive efficiency tasks (64). Moreover, physical activity promotes improvement in children's positive emotions (65), and the total and longest gaze times of subjects with positive emotions are also the greatest (66).

Studies have shown that physical activity (physical exertion, PE) has no effect on children's attention performance (67). Among the factors affecting attention, fundamental movement skills may be the most important influencing factor. The development of fundamental movement skills promotes cognition, and cognitive engagement (CE) in physical activities; moreover, it is the key to improving attention. As CE is a key factor, children's attention significantly improves after motor skill practice exercises 44. The intervention of brief daily bilateral coordination activities for fifth-grade students improved students’ attention and concentration after 4 weeks (68). This research supports the theory of fundamental movement skill development. It is believed that the development of fundamental movement skills in children is the carrier and the basis for the development of cognition and the nervous system. Similarly, physical activity in children and adolescents is positively correlated with overall cognitive function (effect size = 0.21; 8–10 years) (69).

In addition, teachers can effectively guide students in the classroom in terms of skill learning in a relatively short period, and those students who participated in classroom-based physical activity that incorporated skill learning showed significant improvements in task attention (increased 8.3%) (70). Studies have shown a small to moderate improvement in attention to task focus following a break from physical activity (effect size 0.13–0.60) (70). Cognitively engaging in physical activities (such as motor skill learning) is more beneficial for attention improvement (15, 46, 71). CE refers to the allocation of attention and the effort required to participate in an activity (46, 72). Physical activities with higher CE (e.g., playing tennis) had a greater impact on improved attention than did those with low cognitive involvement (e.g., distance running, similar to sports involving more automation) (46, 71). Budde et al. reported that even a set of 10-min coordination exercises (fundamental movement skills exercises) can improve attention in children.

Although this study confirmed the mediating role of fundamental movement skills, related studies have also shown that the impact of physical activity on attention is affected not only by fundamental movement skills but also by aerobic fitness. Related studies have shown that aerobic exercise has the potential to promote attention in children (73). After high-intensity aerobic exercise, frontal cortex activity increases, but temporal cortex activity does not change (74). Research on improving attention in children and adolescents has shown that aerobic exercise is more effective (75), and some studies also demonstrate that short-term aerobic exercise at 60% VO2max can increase cerebral blood flow in the frontal lobe to enhance human attention (76). Sustained aerobic exercise promotes neurological development, accounting for better overall sustained attention over time and a better ability to allocate attentional resources (77). Improvements in aerobic fitness have been shown to improve concentration in an exercise intervention program completed over several weeks (78). In addition, better aerobic fitness in adolescents was associated with greater bilateral hippocampal volume, which was positively correlated with attention in adolescents (79). Adolescents with high aerobic fitness also had larger dorsal striatum volumes, which are associated with cognitive control (80). Moderate- to vigorous-intensity exercise directly and positively impacts attention (81). From the perspective of psychology and physiology, continuous physical activity increases levels of brain neurotrophic factors and causes neurotransmitters (such as adrenaline and dopamine) to promote the improvement in children's attention (82, 83). A possible reason for this outcome is that physical activity promotes the development of fundamental movement skills, and good fundamental movement skills are conducive to engaging in vigorous physical activity that includes aerobic exercise. Higher levels of BDNF improve one's cognitive abilities, thereby improving the physiological excitement level of the human body and promoting attention.

Physical activity affects attention through chain mediation of fundamental movement skills and aerobic fitness. This may be because physical activity promotes improvement in fundamental movement skills, and the development of fundamental movement skills promotes improvement in aerobic fitness (84, 85). Moreover, the development of fundamental movement skills requires cognitive functions, thus improving the distribution of attentional resources (86, 87). This process helps to activate the central nervous system and promote its development to enhance cognitive abilities (78).

Pesce et al. (42) performed related tests of aerobic fitness training or team movement skill-guided aerobic fitness in 11- to 12-year-old subjects. Both physical fitness and participation in a challenging group of aerobic fitness classes benefited delayed memory (12 min) performance, and the latter intervention also enhanced participants’ immediate memory and the correlation between motor skills and aerobic fitness during physical activity. The intervention of aerobic fitness had the added benefit of cognitive activation of memory storage.

This study simultaneously investigated the effects of fundamental movement skills and aerobic fitness on physical activity and attention and explored the relationship between physical activity and attention by combining questionnaires and tests. Hypothesis 1, hypothesis 2, hypothesis 3, and hypothesis 4 were all verified. Physical activity can affect attention through the chain mediating effect of fundamental movement skills and aerobic fitness; that is, the impact of physical activity on attention is affected by the chain effect of fundamental movement skills and aerobic fitness, which can be understood as the effect of physical activity on a virtuous cycle of fundamental movement skills, aerobic fitness, and focus.

When examining the influence of physical activity on attention, we should integrate fundamental movement skills and aerobic fitness at the same time. The dual-system architecture of aerobic fitness, basic movement skills and aerobic fitness also have a certain sequence. This study expands upon previous research on physical activity and attention, enriches the research field of attention to a certain extent, and serves as an important reference for improving attention in children. This study also reminds us that school physical activities should focus on students’ ability to learn and master basic movement skills. Moreover, the level of aerobic fitness further improves during exercise, which helps students master skills and optimize aerobic fitness enhancement.

This study has several limitations. First, the cross-sectional design of the current study prevents conclusions on the direction of causality of these associations from being drawn. Second, research has demonstrated only the correlation between total physical activity and attention. According to the theory of attention, the mechanism of attention is divided into three components: attention vigilance, attention relief difficulty and attention avoidance. Future research can explore the mechanism of attention. Third, whether the improvement in attention by physical activity is related to the type of exercise and which type of exercise has the best effect need further exploration. Fourth, this study analyses the impact of activities on attention and explores only the role of fundamental movement skills and aerobic fitness; there may be other paths involved. Future research should continue to explore possible mediating variables and their mechanisms of action to provide additional interventions and treatments for adolescents. In addition, more research on improved attention in children is needed.

## 5 Conclusions

The present study revealed a positive association between physical activity and attention among children aged 6–12 years. Specifically, we found that fundamental movement skills and aerobic fitness had chain mediating effects on the relationship between physical activity and attention, suggesting the critical role of fundamental movement skills and aerobic fitness in explaining the association between physical activity and attention among children. The results suggest that developing fundamental movement skills and aerobic fitness to one's attention in physical activity can enhance the beneficial impact of good physical activity on attention quality.

## Data Availability

The original contributions presented in the study are included in the article/Supplementary Material, further inquiries can be directed to the corresponding author.
